# Recurrence of optic nerve neuropathy - a possible side effect of intravenous methylprednisolone pulses treatment

**DOI:** 10.1186/1756-6614-6-S2-A62

**Published:** 2013-04-05

**Authors:** Katarzyna Trautsolt, Piotr Miśkiewicz, A Jabłońska, A Samsel, N Wołczańska, D Kęcik, Antoni Krzeski, Tomasz Bednarczuk

**Affiliations:** 1 Department of Endocrinology and Internal Medicine, Medical University of Warsaw, Warsaw, Poland; 2 Department of Ophthalmology, Medical University of Warsaw, Warsaw, Poland; 3 Department of Otolaryngology, Division of Dentistry, First Faculty of Medicine, Medical University of Warsaw, Warsaw, Poland

## Introduction

Graves' orbitopathy (GO) is the most common cause of exophthalmos. The most severe complication of GO is optic neuropathy. Endogenous hypercortisolism (Cushing syndrome) can also lead to exophthalmos by stimulating the proliferation of retrobulbar adipose tissue. Cases of the optic nerve neuropathy due to the glucocorticoids treatment have not been described, so far.

## Case report

A 31-year-old woman with orbitopathy due to Graves' disease, after radioiodine treatment, treated for hypothyroidism with sufficient dose of levothyroxine, was referred to the Department of Endocrinology due to the progressive deterioration of visual acuity and impaired right-eye colour vision. This symptoms have developed for the last two months. On admission, there were features of active orbitopathy (5/7 points on CAS scale), permanent double vision, without exophthalmos. MRI examination revealed active inflammatory process, proliferation of retrobulbar fat, without thickening of oculomotor muscles. Diagnosis of optic nerve neuropathy was established and the patient was treated with high-doses of intravenous (iv) methylprednisolone (MP) pulses - 1g for three consecutive days. After this treatment visual acuity did not improve. The decision of performing urgent bilateral endoscopic decompression was made. The surgical operation restored full visual acuity and colour vision. The clinical features of active inflammatory process receded. Diplopia was still present but only when the patient was looking to the left. The decompression surgery was followed by weekly administration of iv MP pulses (6x500mg, 6x250mg). During the treatment, after the eighth pulse, symptoms of optic nerve neuropathy relapsed in the form of sudden aggravation of visual acuity and impaired colour vision. The clinical activity score remained low (1-2/7). The second bilateral, intranasal, endoscopic decompression was performed, resulting in gradual return of full sharpness of vision. A probable cause of the recurrence of neuropathy symptoms was the proliferation of adipose tissue as a complication of treatment with MP.

## Conclusions

High dose iv MP pulse therapy is a highly effective immunosuppressive treatment used in various inflammatory and autoimmune diseases e.g. active, moderate to severe GO and neuropathy due to GO. It remains to be proven, whether in some cases, the administration of MP can cause orbitopathy as well as may lead to the deterioration of its severity, including optic nerve neuropathy, probably as a result of the proliferation of adipose tissue.

